# Functional and Aesthetic Osseous Free Flap Mandible Reconstruction Using a Low-cost Method: Long-term Results

**DOI:** 10.1097/GOX.0000000000002465

**Published:** 2019-11-12

**Authors:** Carlos Olvera-Caballero, Sergio Cortes-Aroche, Luis G. Vazquez- de- Lara

**Affiliations:** From the * Plastic and Reconstructive Surgery Service, Hospital Ángeles Puebla; † Maxillofacial Surgery Service, Hospital Ángeles Puebla; ‡ Laboratory of Experimental Medicine, School of Medicine, Benemérita Universidad Autónoma de Puebla.

## Abstract

Mandibular segment loss due to tumoral resection or infectious sequelae causes functional alterations with disorders in mastication, swallowing, speech, and aesthetic alterations with facial asymmetry due to deformity secondary to soft tissue collapse. Reconstructive treatment should recover function and aesthetics. This paper presents a low-cost method of mandibular reconstruction in a series of 6 patients with different years of follow-up (average follow-up time of 11.6 years) using the fibula and scapula free flaps.

**Methods:**

Five female patients and 1 male patient received mandibular reconstruction using osseous free flaps, 5 with the fibula and 1 with the scapula osseous free flap. The patient’s ages at the time of surgery ranged from 8 to 62 years (mean 33.1 years). Stainless steel wire was used as the osteosynthesis material, with intermaxillary fixation for 40 days postoperatively and masticatory rehabilitation using mucodental-supported prostheses.

**Results:**

To evaluate the aesthetic result and the facial symmetry, a questionnaire and the photographs of all the cases were sent to 8 plastic surgeons. The functional result was evaluated in 5 of the 6 patients using the Spanish version of the Oral Health Impact Profile. All flaps survived, dental occlusion was achieved in all patients, no tumors recurred, masticatory function was normal without swallowing or speech alterations, and the transplanted bone hypertrophied and spontaneously remodeled, providing facial symmetry with good aesthetic results.

**Conclusion:**

We present a low-cost and universally applicable mandibular reconstruction method, with long-term follow-up and good aesthetic and functional results.

## INTRODUCTION

Mandibular segment loss results in functional and aesthetic disorders. Functional disorders include alterations in mastication, swallowing, and speech, and aesthetic disorders include disfigurement secondary to bone loss with soft tissue collapse. Thus, mandibular reconstruction should aim to restore function and aesthetics.

A balance must be achieved between the masticatory muscles and the bone structure to preserve function. The transplanted bone must be integrated into the residual skeleton and be strong enough to prevent fracture when mandibular function requires it.

Aesthetically, the primary objective must be focused on facial symmetry, which is achieved when the transplanted bone segment provides the shape of the lost bone, thus restoring the mandible’s angles and curves.

The osseous free flaps used in mandibular reconstruction are mainly the fibula, iliac crest, scapula, and radius, which have been widely described.^1–5^

The fibula free flap, because of its easy dissection characteristics, vessels of adequate caliber, good bone length, and possibility of redirecting osteotomies without altering its vascularity, is a good choice in microsurgical reconstruction. The scapula is indicated mainly when extra tissue coverage is required.

Herein, we present a low-cost method of mandibular reconstruction in a series of 6 patients with different years of follow-up (average follow-up time of 11.6 years) using the fibula and scapula free flaps, stainless steel wire as the osteosynthesis material, postoperative intermaxillary fixation for 40 days, and conventional dental rehabilitation with mucodental-supported prostheses.

We demonstrated spontaneous remodeling and hypertrophy of the transplanted bone with normal masticatory function, no swallowing or speech alterations, and satisfactory aesthetic facial symmetry results.

## MATERIALS AND METHODS

According to the classification system of Rodriguez et al,^6^ the defects corresponded to 2 type 1 A defects, 3 type 2 A defects, and 1 type 3 defect; 6 cases are summarized in Table 1.

**Table 1.**
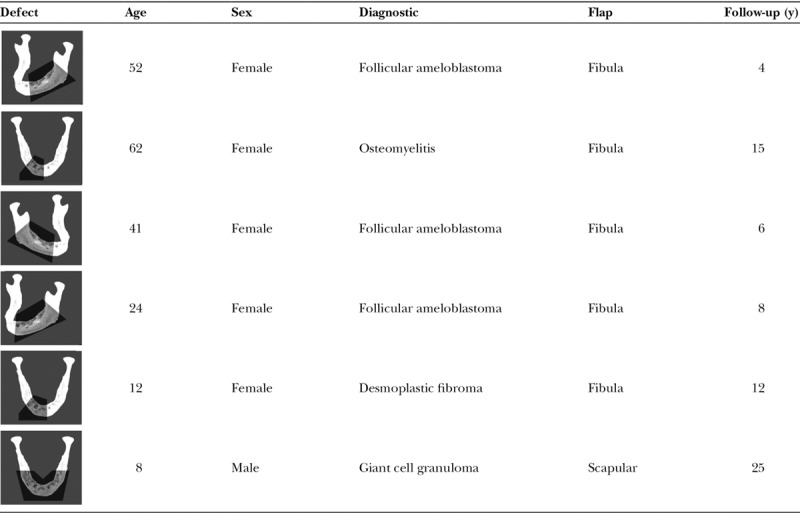
Six Cases of Mandibular Reconstruction

Surgery was planned based on radiographic studies. The ipsilateral fibula was used in 5 patients, and the lateral border of the left scapula was used in 1 patient. End-to-end microvascular anastomoses were performed using 9-0 nylon between the peroneal artery and vein and the scapular circumflex artery and vein with the corresponding facial artery and vein.

Patients received nasotracheal intubation, and arch splints were placed before starting the surgery for intermaxillary fixation during the postoperative period. Only an arch splint was placed in the male pediatric patient’s maxilla. We worked with 2 surgical teams, 1 in the head and other in the leg or in the back.

To remove the tumor, a cutaneous incision was made below the lower mandibular border. The osseous segment was removed with borders from the healthy bone to serve as a model for measuring and redirecting the partial osteotomies that were performed in the 6 flaps on the auxiliary table. Subsequently, the free flap on the mandible was stabilized, and end-to-end microvascular anastomoses were performed using 9-0 nylon between the peroneal artery and vein and the scapular circumflex artery and vein with the corresponding facial artery and vein.

Osteosynthesis of the osseous free flap to the mandible was performed using stainless steel wire, placing the transplanted bone segment in close contact with the mandibular bone and as close as possible to the lower mandibular border. The intermaxillary fixation was done with bands 24 hours after surgery and then with wire after the first 72 hours and maintained for 40 days. In the male pediatric patient, the intermaxillary fixation was done between the maxillary arch splint and the mandibular angles with wire fixed to a cortical screw that were removed at 40th postoperative day.

The patients were initially fed a liquid diet and then a pureed diet with a straw. The area of the osseous free flap, being devoid of teeth, allowed food to pass.

During the postoperative period, patients received dextran as a platelet antiaggregant, broad-spectrum antimicrobials, analgesics, and life support. The hospitalization time averaged 1 week. Radiographic controls were performed at 5, 8, and 12 weeks and afterward by scheduled visits. The average follow-up time was 11.6 years (4–25 years).

The aesthetic result was measured with a 5-item ad hoc questionnaire (Table 2). The score consisted of a Likert-type scale from 0 to 4, arranged in such a way that 0 means the poorest and 4 the most adequate aesthetic outcome. Eight certified plastic surgeons evaluated the photographs of the 6 patients. Interrater reliability was measured with the “raters” package of the software R (version 3.5.2); it is a modification of Fleiss’ Kappa for nominal and ordinal variables.^7^

**Table 2.** Five-item Questionnaire Designed to Assess the Aesthetic Result

On a scale from 0 to 4, where 0 is “completely agree” and 4 is “completely disagree”, evaluate the following questions after checking the attached file containing the images of each of the 6 patients who form this study group
1. Observe facial symmetry in this patient with mandibular reconstruction
2. Think that the appearance of the patient can condition social isolation
3. Find the patient with facial symmetry according to their ethnic characteristics
4. Consider that the remodeling and spontaneous hypertrophy of the bone has conditioned facial symmetry
5. Considering the previous tumor, I think that the reconstruction made provides the patient with a good aesthetic result

To assess the functional results, the Spanish Version of the Oral Health Impact Profile (OHIP-Sp) was used.^8,9^ It consists of 49 items which evaluate 7 dimensions of impact: functional limitation, physical pain, psychological discomfort, physical disability, psychological disability, social disability, and handicap. Respondents answer how frequently had experienced each problem in the past 12 months on a 5-point Likert-type scale. Response categories are 0 (never or not applicable), 1 (hardly ever), 2 (occasionally), 3 (fairly often), and 4 (very often). OHIP-Sp scores were computed counting, for each subject, the number of items reported at each category.

## RESULTS

All flaps survived with no postoperative major complications, and the oral mucosa healed without problems, except in the pediatric male patient who required secondary suture. The process of osseous consolidation of the free flap to the mandible was observed on the radiographs at 40 days postoperatively when the intermaxillary fixation was withdrawn. Dental occlusion was satisfactory in all patients. No tumor relapses or sequelae occurred in the donor areas, and no patients experienced swallowing or speech alterations.

**Fig. 1.**
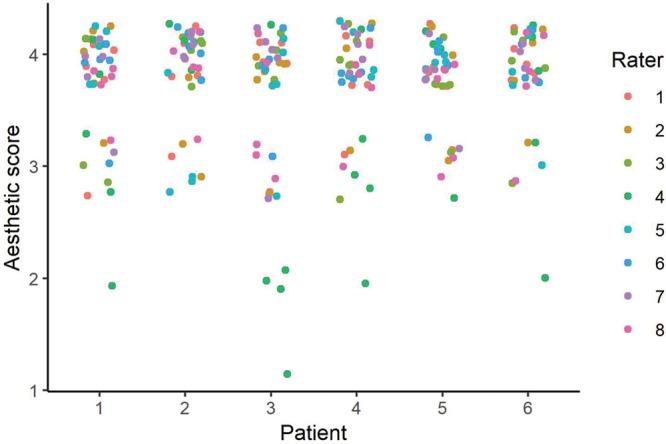
Evaluation of the aesthetic results with an ad hoc questionnaire. Eight certified plastic surgeons independently evaluated the photographs of the 6 patients (raters). The score ranged from 0 to 4 (poorest to most adequate outcome).

The evaluation of the aesthetic result is depicted in Figure 1 for each patient. The interrater agreement was 0.71, confidence interval_95%_ (0.63–0.79). Overall, the median of the evaluation by the 8 raters was 4, which is the highest score.

The results of the functional evaluation for each patient are summarized in Table 3. The OHIP-Sp questionnaire was sent to 5 of the 6 patients. Overall, 93% of the items were categorized between “never” and “hardly ever,” which can be interpreted as an excellent functional outcome.

**Table 3.** Results of the Self-reported OHIP-Sp Instrument in 5 Patients

Patient	Response Categories (%)			
	Never	Hardly Ever	Occasionally	Fairly Often
1	77.6	22.4	0	0
2	75.5	24.5	0	0
3	85.7	14.3	0	0
4	55.1	26.5	12.2	6.1
5	65.3	18.4	10.2	6.1
Total	71.8	21.2	4.5	2.4

Overall percentage of responses in each category.

Hypertrophy and spontaneous remodeling of the transplanted bone supporting the soft tissues occurred in all cases which is shown in the video images accompanying this paper. (See Video, [online], which shows 6 cases of mandibular reconstruction with preoperative, intraoperative, and postoperative clinical and radiographic images.)

**Video.** This video shows the six cases of mandibular reconstruction with preoperative, intraoperative and postoperative clinical and radiographic images.

Masticatory function was normal, and bite was restored using mucodental-supported prostheses.

## DISCUSSION

Reconstructive microsurgery using free flaps has advanced from being a technique to replace and/or cover areas of tissue loss with exposure of vital structures to the aggregation of an aesthetic component. When possible, flaps are selected that, when placed in a specific site, leave the least aesthetic sequelae with less bulging and better adaptation to the receiving site.

Mandibular reconstruction is challenging, and recovery of function and facial aesthetics go hand in hand. Osseous free flaps must be planned with that intention.^6,10^

Virtual surgical planning has been introduced in recent years as an auxiliary method for mandibular reconstruction with osseous free flaps, where, using stereolithographic models, osteotomy guides, and prefabricated titanium plates, surgeons try to improve precision and decrease operative time. However, use of this technology, its universal application, and its long-term results have not been fully evaluated, but simplification, cost reduction, and widespread use are expected with these technological advances.^11,12^

In the past, we used the iliac crest osseous free flap originally described by Taylor, which can be modeled to the shape of the original mandible. However, we faced a more complicated dissection with a short vascular pedicle and the possibility of abdominal hernias at the donor site.^13^

The fibula free flap has advantages because of its relatively simple dissection, the possibility of performing redirective osteotomies without harming the vascularity, and its long vascular pedicle adapts well to the mandible. Sequelae in the donor area, when not used with a skin island, are nearly nonexistent. The scapular flap is mainly indicated when extra soft tissue is required. In our case, the scapular flap was chosen because the patient’s parents requested that we not use the lower extremities as a donor area.^14^ As with any bone transplant, a resistant osseous callus must be formed to achieve a functional result, and the immobility of the osseous unions is essential in the cicatricial aspect; hence, fixation with osteosynthesis material is always necessary.

Titanium plates are widely used as an osteosynthesis material in mandibular reconstruction and provide suitable stability for the neomandible, although they may induce complications, such as detriment to the vascularity of the transplanted bone by the fixation screws and possible extrusion or fracture.^15^ In addition, we think that they partially support the masticatory forces limiting hypertrophy and the spontaneous remodeling of bone and soft tissues.

In these cases, osteosynthesis was performed using stainless steel wire, mainly because of its availability and its low cost compared with prefabricated titanium plates. Besides, this material does not harm the vascularity of the transplanted bone.

We observed no complications of pseudoarthrosis in the osteosynthesis sites with the mandible or in the areas of redirected osteotomies.

**Fig. 2.**
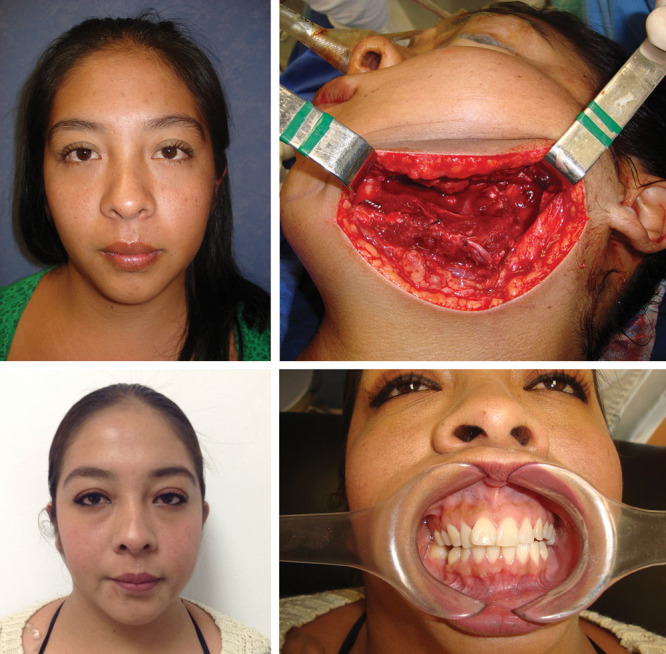
Patient number 4. Upper row, from left to right: preoperative frontal view; left follicular ameloblastoma; intraoperative view of the fibula free flap with wire osteosynthesis; 8-year postoperative view; facial symmetry; occlusion view.

**Fig. 3.**
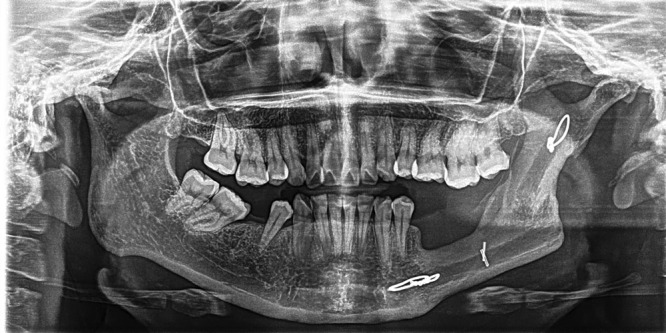
Orthopantomography at 8 years showing spontaneous bone remodeling.

The wire also allows the transplanted bone to hypertrophy and spontaneously remodel, which is functionally necessary because it supports the masticatory force, and simultaneously, hypertrophy and spontaneous remodeling provide a good aesthetic result by symmetrizing the face. In addition, removal of the wire after bone healing is not needed, we have not observed fractures, and neither extrusion nor body reaction to this material as it can be seen in the x-ray postoperative images (Figs. 2 and 3 and see Video [online], which shows 6 cases of mandibular reconstruction with preoperative, intraoperative, and postoperative clinical and radiographic images).

The 40-day intermaxillary fixation represents further discomfort for patients during the postoperative period. However, we believe the results justify its use.

Dental rehabilitation was performed by placing mucodental-supported prostheses. Although patients could have had dental implants placed, the patients chose not to undergo more surgeries either for economic reasons or because their mastication was normal with the removable prostheses.

Finally, we would like to mention the drawbacks of this work both in its design and in the proposed technique of mandibular reconstruction. Given the small number of patients, the variability in the follow-up time and that a single technique was not used in all patients, the extrapolation of the results has limitations that must be taken into account. It is also worth noting that this technique does not represent the standard of treatment currently accepted for mandibular reconstruction, in which virtual surgical planning with stereolithographic models, osteotomy guides, and prefabricated titanium plates provide more predictable results. Even though we did not have complications related to the material used, we are aware that the wire used in the osteosynthesis can fail and even extrude, complicating bone healing. This technique can be used, following the guidelines established in this work, in places where the most expensive reconstruction techniques are not available.

## CONCLUSIONS

The low-cost method of mandibular reconstruction with osseous free flaps presented here, using stainless steel wire as an osteosynthesis material, postoperative intermaxillary fixation plus conventional dental rehabilitation with mucodental-supported prostheses provides, in the long term, acceptable functional and aesthetic results with hypertrophy of bone and soft tissue remodeling in both children and adults.

## References

[1] Zenn MR Hidalgo DA Cordeiro PG Current role of the radial forearm free flap in mandibular reconstruction. Plast Reconstr Surg. 1997;99:1012–1017. 9091896 10.1097/00006534-199704000-00014

[2] Taylor GI Reconstruction of the mandible with free composite iliac bone grafts. Ann Plast Surg. 1982;9:361–376. 6758673 10.1097/00000637-198211000-00003

[3] Johnson TE Bayles SW Burkey BB Renewed popularity of scapular osteocutaneous free flaps for complex head and neck reconstruction. Curr Opin Otolaryngol Head Neck Surg 2002;10:261–265.

[4] Hidalgo DA Pusic AL Free-flap mandibular reconstruction: a 10-year follow-up study. Plast Reconstr Surg. 2002;110:438–449; discussion 450. 12142657 10.1097/00006534-200208000-00010

[5] Hidalgo DA Fibula free flap: a new method of mandible reconstruction. Plast Reconstr Surg. 1989;84:71–79. 2734406

[6] Schultz BD Sosin M Nam A Classification of mandible defects and algorithm for microvascular reconstruction. Plast Reconstr Surg. 2015;135:743e–754e. 25811586 10.1097/PRS.0000000000001106

[7] Marasini D Quatto P Ripamonti E Assessing the inter-rater agreement for ordinal data through weighted indexes. Stat Methods Med Res. 2016;25:2611–2633. 24740999 10.1177/0962280214529560

[8] Slade GD Spencer AJ Development and evaluation of the oral health impact profile. Community Dent Health. 1994;11:3–11. 8193981

[9] Lopez R Baelum V Spanish version of the oral health impact profile (OHIP-sp). BMC Oral Health. 2006;6:11. 16827940 10.1186/1472-6831-6-11 PMC1534011

[10] Cordeiro PG Henderson PW Matros E A 20-year experience with 202 segmental mandibulectomy defects: a defect classification system, algorithm for flap selection, and surgical outcomes. Plast Reconstr Surg. 2018;141:571e–581e. 10.1097/PRS.0000000000004239 PMC5880317 29596191

[11] Deek NF Wei FC Computer-assisted surgery for segmental mandibular reconstruction with the osteoseptocutaneous fibula flap: can we instigate ideological and technological reforms? Plast Reconstr Surg. 2016;137:963–970. 26910680 10.1097/01.prs.0000479998.49928.71

[12] Chang EI Jenkins MP Patel SA Long-term operative outcomes of preoperative computed tomography-guided virtual surgical planning for osteocutaneous free flap mandible reconstruction. Plast Reconstr Surg. 2016;137:619–623. 26818299 10.1097/01.prs.0000475796.61855.a7

[13] Olvera-Caballero C Cortes-Aroche S Rueda-Alvarado CR Gonzalez-Aguilar H Reconstrucción mandibular con el colgajo libre de cresta iliaca. Cir Plast IberLatin 1988;14:149–155.

[14] Olvera-Caballero C Mandibular reconstruction in children. Microsurgery. 2000;20:158–161. 10980513 10.1002/1098-2752(2000)20:4<158::aid-micr2>3.0.co;2-l

[15] Robey AB Spann ML McAuliff TM Comparison of miniplates and reconstruction plates in fibular flap reconstruction of the mandible. Plast Reconstr Surg. 2008;122:1733–1738. 19050525 10.1097/PRS.0b013e31818a9ac5

